# Regulatory genes in the androgen production, uptake and conversion (APUC) pathway in advanced prostate cancer

**DOI:** 10.1530/EO-22-0058

**Published:** 2022-06-07

**Authors:** Sean McSweeney, Hannah E Bergom, Anna Prizment, Susan Halabi, Nima Sharifi, Charles Ryan, Justin Hwang

**Affiliations:** 1 University of Minnesota Medical School, Minneapolis, Minnesota, USA; 2 Department of Medicine, University of Minnesota Masonic Cancer Center, Minneapolis, Minnesota, USA; 3 Division of Hematology, Oncology and Transplantation, University of Minnesota, Minneapolis, Minnesota, USA; 4 Department of Biostatistics and Bioinformatics, Duke University, Durham, North Carolina, USA; 5 Genitourinary Malignancies Research Center, Lerner Research Institute, Cleveland Clinic, Cleveland, Ohio, USA; 6 Prostate Cancer Foundation, Santa Monica, California, USA

**Keywords:** genomics, prostate cancer, androgens

## Abstract

The androgen receptor (AR) signaling pathway regulates the progression of prostate cancer (PC). Metastatic castration-resistant prostate cancer (mCRPC) patients generally receive AR-targeted therapies (ART) or androgen-deprivation therapies (ADT) with the initial response; however, resistance is inevitably observed. Prior studies have shown activity and upregulation of a family of androgen production, uptake, and conversion – APUC genes – based on genomic analyses of patient germlines. Genetic variants of some APUC genes, such as the conversion gene, HSD3B1, predict response to second-generation androgen-targeted therapies. Studies have begun to elucidate the overall role of APUC genes, each with unique actionable enzymatic activity, in mCRPC patient outcomes. The current role and knowledge of the genetic and genomic features of APUC genes in advanced prostate cancer and beyond are discussed in this review. These studies inform of how interpreting behavior of APUC genes through genomic tools will impact the treatment of advanced prostate cancer.

## Overview of APUC genes

**Figure 1:**
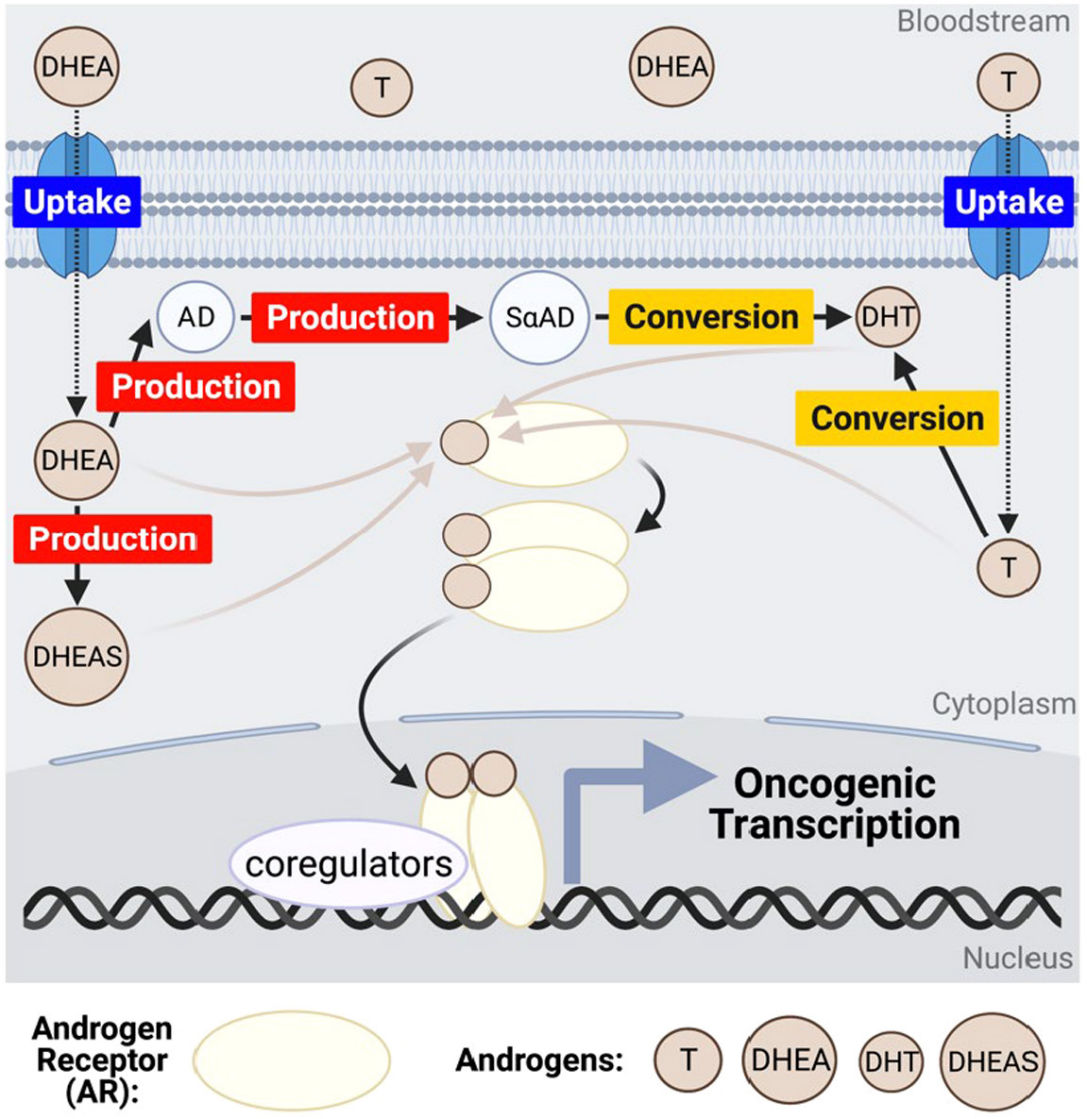
Androgen signaling in the APUC pathway leads to oncogenic transcription. AR, androgen receptor; T, testosterone; DHT, dihydrotestosterone; DHEAS, DHEA sulfate.

Androgen receptor (AR) activation is required for promotion of prostate cancer and remains critical in the development of metastatic castration-resistant prostate cancer (mCRPC). Androgens are the circulating hormone activators of the AR and therefore drive prostate cancer growth and progression in mCRPC patients as well as laboratory prostate cancer cell models. Androgens, such as testosterone and dihydrotestosterone (DHT), are both taken up by the cell and created *de novo* from within the tumor cell from precursor molecules (Fig. 1). However, to optimize dependence on AR, tumors may adopt various mechanisms to regulate genes associated with androgen production, uptake, and conversion – being referred to heretofore as the APUC family of genes. Increasingly, studies have begun to highlight the importance of APUC genes on mortality and cancer-related outcomes in mCRPC patients.

The reliance of prostate cancer cells on androgen signaling has led to an emphasis on inhibiting AR signaling as a pharmacologic treatment, even in castration-resistant disease. This treatment includes current forms of second-generation AR-targeted therapies (ART) via pharmacologic antagonists such as apalutamide, darolutamide, and enzalutamide. Targeting APUC genes in mCRPC is also considered, as the second-generation ART, abiraterone acetate, blocks *de novo* androgen synthesis within tumor cells using cytochrome p450 17 alpha-hydroxylase (CYP17) (Ryan *et al.* 2013). However, resistance inevitably occurs often via mechanisms with diverse features associated with genomic alterations that impact AR cell signaling. Particularly, a subset of the resistant mCRPC harbors AR genomic alterations through either mutations, copy number gain, enhancer amplification or increased resistant transcript variants (AR-V7) (Antonarakis *et al.* 2014, Abida *et al.* 2019, He *et al.* 2021). While these features of mCRPC stress a relevance of AR signaling, there have been significantly less studies that examine APUC genes. However, evidence indicates that genetic or genomic variations in the APUC genes may dysregulate the androgen supply chain in mCRPC and are associated with differences in survival and mortality outcomes (Feldman & Feldman 2001, Almassi *et al.* 2018).

A growing availability of genetic, genomic, and transcriptomic data from metastatic prostate cancer patients now provides additional means to investigate predictors of prostate cancer. This includes diagnostic technology and informatics tools that permit us to examine association of clinical observations with any genetic variation and genomic alteration outside of AR. While age and family history have long been known as the strongest predictors of prostate cancer, genomics tools have discerned that prostate cancer harbors distinct mechanism of tumorigenesis within certain ethnic populations (Mahal *et al.* 2020). Already, prognostication and treatment decision tools are useful in the way of DECIPHER testing (Karnes *et al.* 2013, Angeles *et al.* 2018). Logically, these genetic and genomic tools permit the examination of APUC genes as factors that regulate disease progression of prostate cancer patients. However, while individual APUC genes have been examined, only few studies (Prizment *et al.* 2021) have considered them in aggregate or as a part of a larger network of a specific androgen production, uptake, and conversion signaling pathway.

Emerging literature is rapidly expanding our understanding of the clinical ramifications of APUC gene perturbations within prostate cancer and other endocrine- and steroid-driven tumors. Based on literature review, we have identified and honed in on 21 APUC genes shown in the literature to have genetic variations in prostate cancer (Table 1). These genes were grouped based on their enzymatic activity into either the production, uptake, or conversion category. Here we summarize prior research studies that have pioneered our current understanding of APUC genes.

**Table 1:** A curated list of APUC genes studied in prostate cancer.

Official gene name	Production/uptake/conversion	Approved symbol	Approved name	HGNC ID	Location	Reference
AKR1C3	Production	AKR1C3	Aldo-keto reductase family 1 member C3	HGNC:386	10p15.1	Nyquist *et al.* 2019
CYP1A1	Conversion	CYP1A1	Cytochrome P450 family 1 subfamily A member 1	HGNC:2595	15q24.1	Li *et al.* 2012
CYP1B1	Conversion	CYP1B1	Cytochrome P450 family 1 subfamily B member 1	HGNC:2597	2p22.2	Li *et al.* 2012
CYP3A43	Conversion	CYP3A43	Cytochrome P450 family 3 subfamily A member 43	HGNC:17450	7q22.1	Han *et al.* 2015
CYP11A1	Production	CYP11A1	Cytochrome P450 family 11 subfamily A member 1	HGNC:2590	15q24.1	Fan *et al.* 2016 Oki *et al.* 2021
CYP11B1	Production	CYP11B1	Cytochrome P450 family 11 subfamily B member 1	HGNC:2591	8q24.3	Fan *et al.* 2016 Oki *et al.* 2021
CYP17A1	Production	CYP17A1	Cytochrome P450 family 17 subfamily A member 1	HGNC:2593	10q24.32	Yamada *et al.* 2013 Robles-Fernandez *et al.* 2017 Wright *et al.* 2020
CYP19A1	Conversion	CYP19A1	Cytochrome P450 family 19 subfamily A member 1	HGNC:2594	15q21.2	Travis *et al.* 2009 Kanda *et al.* 2015
HSD3B1	Conversion	HSD3B1	Hydroxy-delta-5-steroid dehydrogenase, 3 beta- and steroid delta-isomerase 1	HGNC:5217	1p12	Wright *et al.* 2020
HSD3B2	Conversion	HSD3B2	Hydroxy-delta-5-steroid dehydrogenase, 3 beta- and steroid delta-isomerase 2	HGNC:5218	1p12	Wright *et al.* 2020
HSD17B3	Conversion	HSD17B3	Hydroxysteroid 17-beta dehydrogenase 3	HGNC:5212	9q22.32	Nyquist *et al.* 2019
HSD17B6	Conversion	HSD17B6	Hydroxysteroid 17-beta dehydrogenase 6	HGNC:23316	12q13.3	Nyquist *et al.* 2019
HSD17B10	Conversion	HSD17B10	Hydroxysteroid 17-beta dehydrogenase 10	HGNC:4800	Xp11.22	Nyquist *et al.* 2019
LHCGR	Uptake	LHCGR	Luteinizing hormone/choriogonadotropin receptor	HGNC:6585	2p16.3	Xiong *et al.* 2015
SLCO2B1	Uptake	SLCO2B1	Solute carrier organic anion transporter family member 2B1	HGNC:10962	11q13.4	Wright *et al.* 2011
SLCO1B3	Uptake	SLCO1B3	Solute carrier organic anion transporter family member 1B3	HGNC:10961	12p12.2	Wright *et al.* 2011
SRD5A1	Production	SRD5A1	Steroid 5 alpha-reductase 1	HGNC:11284	5p15.31	Nyquist *et al.* 2019 Wright *et al.* 2020
SRD5A2	Conversion	SRD5A2	Steroid 5 alpha-reductase 2	HGNC:11285	2p23.1	Nyquist *et al.* 2019 Wright *et al.* 2020
SRD5A3	Production	SRD5A3	Steroid 5 alpha-reductase 3	HGNC:25812	4q12	Nyquist *et al.* 2019 Wright *et al.* 2020
SULT2A1	Production	SULT2A1	Sulfotransferase family 2A member 1	HGNC:11458	19q13.33	Wilborn *et al.* 2006
SULT2B1	Conversion	SULT2B1	Sulfotransferase family 2B member 1	HGNC:11459	19q13.33	Nyquist *et al.* 2019

## Clinical outcomes of *HSD3B1* genetic alterations in prostate cancer

The most widely studied APUC enzyme is 3β-hydroxy­steroid dehydrogenase-1 encoded by the gene *HSD3B1*. Expression of 3βHSD1 is found primarily in the peripheral non-endocrine tissues of the body and catalyzes the conversion of DHEA into androstenedione, which is then used as a substrate to create the potent AR-activating agent DHT. This enzyme is required for the production of all other non-testicular testosterone or DHT (Thomas *et al.* 2020). This role and the subsequent ability to create DHT precursors in the adrenal glands becomes important in the context of mCRPC where the prostate cancer has found a role to grow in the setting of no other endogenous androgen production due to various forms of ADT/ART. The role of *HSD3B1* in the outcome of prostate cancer patients is an important landmark and example that a single APUC gene impacts the patient’s survival. Here we will look at the significant data on this one APUC gene to this point and the role of *HSD3B1* in other endocrine tumors.

The role of 3βHSD1 variation in prostate cancer arises predominantly from a germline missense-encoding variant (1245A→C) of the gene *HSD3B1*. This allelic variant renders the 3βHSD1 protein more stable and resistant to ubiquitination and degradation. This increased stability allows for increased levels of 3βHSD1 and higher levels of potent downstream androgens such as DHT that can go on to activate AR-sensitive tissues such as prostate and prostate cancers (Chang *et al.* 2013). Previous work has named the *HSD3B1* (1245C) allele the ‘adrenal permissive’ type as it creates a phenotype that causes increased rates of adrenal potent androgen synthesis and conversely have named the *HSD3B1* (1245A) allele the ‘adrenal restrictive’ type as this genotype causes decreased generation of potent adrenal androgens (Sabharwal & Sharifi 2019, Naelitz & Sharifi 2020).

While Sharifi *et al.* have identified the presence of *HSD3B1* allelic variants in prostate cancer patients and how this may yield differing levels of downstream androgens, other works have further identified differences in mortality and outcomes of such patients in the context of allelic variants. Initial studies examined the role of *HSD3B1* allelic variations in men with hormone-refractory prostate cancer. In two separate studies at Cleveland Clinic and Mayo Clinic, patients who had undergone definitive prostate cancer treatment via either radical prostatectomy or radiation therapy and were subsequently started on ADT for biochemical recurrence and contained the adrenal permissive *HSD3B1* (1245C) allele had worse rates of progression-free, metastasis-free, and overall survival from prostate cancer. These results also showed that the rates of overall survival, progression-free survival, and metastasis-free survival were worse in a dose–response curve type manner with increasing rates of the adrenal permissive allele number (Hearn *et al.* 2016).

*HSD3B1* allelic variations also impact outcomes of metastatic castration-sensitive prostate cancer (mCSPC) patients treated with ADT. Agarwal *et al.* examined the rates of progression-free survival in 102 mCSPC patients treated with ADT and found that patients homozygous for the adrenal permissive allele (11 months) exhibited worse outcomes as compared to ones with the adrenal restrictive allele (21 months) (Agarwal *et al.* 2017). In another study of 104 Japanese men with mCSPC being treated with ADT, progression-free survival was significantly decreased in men with at least one adrenal permissive allele compared to the ones homozygous for the adrenal-restrictive allele (Shiota *et al.* 2019). These two studies were conducted in two populations of distinct ethnicities that were associated with distinct baseline allelic variation rates. Specifically, the Japanese study had far lesser rates of the adrenal-permissive allele (14.1%) than in the other studies with a largely White cohort (26–36%) (Agarwal *et al.* 2017, Shiota *et al.* 2019), yet these significant differences in progression-free survival were still observed. Additionally, the role of these *HSD3B1* allelic variations has been studied in metastasis-free survival after initiation of ADT. In a cohort of patients given ADT for biochemical recurrence following definitive treatment with radiation therapy, there was a significantly shorter metastasis-free survival in patients either homozygous or heterozygous for the adrenal-permissive alleles vs those homozygous for adrenal-restrictive alleles (5.8 years and 4.4 years for 1 and 2 adrenal-permissive alleles, respectively, vs 7.4 years for 0 alleles) (Hearn *et al.* 2018). Together, these studies reveal that *HSD3B1* allelic variations are associated with difference in outcomes in both non-metastatic castration sensitive prostate cancer (nmCSPC) and mCSPC patients being treated with ADT. Mechanistically, the adrenal-permissive *HSD3B1* allelic status may regulate either the tumor or be conditioning its microenvironment and in turn supporting resistance to ADT and subsequently developing worse survival outcomes at increased rates.

Studies have examined the role of these allelic variations in men treated with both ADT and another chemotherapeutic agent. Hearn *et al.* examined data from mCSPC patients enrolled in the Chemohormonal Therapy versus Androgen Ablation Randomized Trial for Extensive Disease in Prostate Cancer (CHAARTED) study examining survival metrics in mCSPC patients treated with either ADT alone or ADT plus docetaxel. They found no difference in survival outcomes among patients with different allelic variations *HSD3B1* treated with ADT plus docetaxel or ADT alone. When they stratified for low- vs high-volume disease, the adrenal-permissive allele was not associated with worse outcomes in patients with high-volume disease; however, interestingly, they observed decreased susceptibility of CRPC after 2 years in patients with low-volume disease. The conclusions of this study also indicated that higher disease burden is probably associated with increased global genomic alterations of other tumor regulatory genes. These additional genomic alterations in other potential genes or mechanisms may support growth of tumors that were less reliant on extra-gonadal androgen production (such as that conferred by the adrenal-permissive *HSD3B1* allele) (Hearn *et al.* 2020).

Other studies have further examined the role of *HSD3B1* allelic variations in mCRPC. Almassi *et al.* found that within men with mCRPC who were being treated with ketoconazole which is a non-steroidal CYP17A1 and CYP11A1 inhibitor that blocks the adrenal production of androgens, there was a longer period of progression-free survival in men who contained at least one allele of the adrenal-permissive *HSD3B1* (Almassi *et al.* 2018). These findings further indicate that mCRPC is at least partially dependent on extra-gonadal androgen production.

Altogether, these findings stress the importance of delineating which mCSPC or mCRPC patients possess one or multiple copies of the adrenal-permissive *HSD3B1*. In addition to ADT, the overall genetics of HSD3B1 also informs of differences in dependency of extra-gonadal androgens, which supports treatment decisions using next-generation chemotherapies or non-hormonal chemotherapies.

## Prostate cancer tumor genomics associated with *HSD3B1* variations

In addition to the large body of literature examining the role of *HSD3B1* genetics, work has been done to examine somatic alterations of *HSD3B1* in prostate cancer through genomic tools. Notably, Shiota *et al.* performed Sanger sequencing from whole blood sampling, commercial prostate cancer cell lines, as well as both metastatic lymph node tissue and tissue from primary prostate tumors of mCRPC patients on ADT. They found not only somatic mutations in *HSD3B1* in multiple samples but also amplification and overexpression of *HSD3B1* within prostate cancer cell lines and tissues from mCRPC patients. Their findings corroborated the adrenal-permissive genotype of *HSD3B1* association with increased risk of cancer progression within high-volume disease but not low-volume disease in these mCRPC patients (Shiota *et al.* 2022).

In 101 mCRPC patients on ADT, Chen *et al.* analyzed germline polymorphisms of 9 androgen synthesis germline variants including in the adrenal-permissive allele of *HSD3B1*. Upon considering RNA and DNA sequenced tumors, they conducted informatics analyses to associate genetic variant status with patient outcomes, putative prostate cancer genomic features, and transcriptional profiles (Chen *et al.* 2020). Upon examine RNA-sequencing profiles, samples harboring the adrenal-permissive *HSD3B1* were associated with overexpression of genes belonging to ten pathways representing increases of cell-cycle and tumor proliferation. Thus, germline* HSD3B1* variant status could predict the activity of the cell-cycle pathway as well as molecularly and clinically aggressive tumors. Their corroborated results showed that the adrenal-permissive *HSD3B1* (1245A→C) was associated with a decreased overall survival; however, the germline variations in *HSD3B1* were not significantly associated with putative somatic tumor DNA alterations, including the tumor suppressor gene *RB1*. It has been independently shown that loss of the tumor suppressor gene *RB1* is associated with decreased overall survival in men with mCRPC (Chen *et al.* 2019), regardless of their exposure to either abiraterone or enzalutamide (Abida *et al.* 2019). *RB1* is an important tumor suppressor gene involved in regulating DNA replication prior to cell division (Mulligan & Jacks 1998, Goodrich 2006) and thus acts as a key regulatory of cell-cycle pathways. The multivariate analysis performed by Chen *et al.* also examined the relationship between *RB1* loss or *HSD3B1* variant status as a function of overall survival and clinicopathologic mCRPC features. Here, they found that both were independently predictive of shorter overall survival time after initiation of ADT (Chen *et al.* 2020). These results indicate that *HSD3B1* variants and somatic *RB1* loss act as two independent pathways, in which either germline or somatic changes could be used to predict outcomes of the same cohort of mCRPC patients*.* However, additional studies in larger cohorts with diverse patient populations are warranted to support these conclusions.

## *HSD3B1* outside prostate cancer

APUC genes have also been studied within the context of other endocrine- and hormone-driven tumors. It has been shown that breast cancer patients with SNPs in multiple APUC genes have different levels of both estradiol and testosterone concentrations within breast tissue (Lee *et al.* 2022). *HSD3B1* and its genetic variations play a role in breast cancer. At baseline, the 3βHSD1 enzyme catalyzes the conversion of steroid hormones such as pregnenolone and DHEA into more potent AR- and ER-active steroid hormones such as progesterone, testosterone, and androstenedione (Puddefoot *et al.* 2006). Testosterone and androstenedione can be further converted into ER-activating estrogens by aromatase, which then go on to drive breast cancer growth and development (Roy & Vadlamudi 2012). Research has been done examining the use of trilostane, a 3βHSD1 inhibitor, in the treatment of breast cancer and has been shown in clinical trials to be an effective treatment in individuals with recurrence of their disease while on at least one other form of anti-estrogen treatment (Puddefoot *et al.* 2006). Liu *et al.* examined over 250 breast cancer tumors in tumor tissue arrays and found increased 3βHSD1 protein expression. When they conducted genetic knockdown of *HSD3B1* or applied pharmacologic inhibition of 3βHSD1 with trilostane, they observed attenuation of cellular proliferation and migration of breast cancer cell lines. Furthermore, they demonstrated with *in vivo* studies that trilostane significantly slowed breast cancer tumor growth (Chang *et al.* 2017). In a separate study, the adrenal-permissive *HSD3B1* variant was shown to be associated with increased rates of estrogen-driven postmenopausal breast cancer, given increased levels of circulating androstenedione that can activate the estrogen receptor, and that this genotype was associated with estrogen-driven postmenopausal breast cancer (Kruse *et al.* 2021). This continues to be an active area of research with many forthcoming presentations and papers addressing the role of *HSD3B1* in estrogen-driven malignancies.

Studies have also examined the role of *HSD3B1* and other APUC genes outside of strictly androgen- or estrogen-driven malignancies. Within hepatocellular carcinoma (HCC), studies* in vitro* have recently shown that inhibition of *HSD3B1* with trilostane caused significant inhibition HCC cell clonogenicity and cellular migration. Furthermore, inhibition of *HSD3B1* in combination with the EGFR protein kinase inhibitor Sorafenib significantly inhibited the growth and migration of HCC cells more than either of the two given individually (Lin *et al.* 2021).

Overall, the story on the impact of allelic variations in *HSD3B1* continues to be told across prostate cancers, other endocrine-driven diseases, or even malignancies in which we may not expect. However, it remains important to consider the role and body of evidence behind this one important enzyme and gene in the contexts of other APUC genes. Future work that examines the role of other APUC genes, individually or together, within these disease states provides potential opportunity for deeper understandings of tumor biology and may provide utilities as biomarkers or discover or therapeutic targets.

## Non-HSD3B1 APUC genetic variants

While studies of HSD3B1 have yielded intriguing insights, the host of other APUC genes and their genetic or somatic perturbations have also been examined in prostate cancer and beyond. The story for some of these genes within prostate cancer is not as clear at the moment and will warrant further study. Here, we discuss the known functions for additional APUC genes in prostate cancer.

SNPs within the APUC gene *SLCO2B1*, which encode for the solute carrier organic ion pump, have an interesting story thus far. These have been examined by two studies that presented conflicting results upon examining the same SNPs in relation to prostate cancer outcomes within White men on ADT (Yang *et al.* 2011, Wang *et al.* 2016). Wang *et al.* demonstrated an association of decreased time to progression in patients treated with ADT with exonic SNP rs12422149 (Wang *et al.* 2016), while Yang *et al.* found this association with not only rs12422149 but also in intronic rs1789693 and rs1077858 (Yang *et al.* 2011). Studies by Wang *et al.* further examined overall survival and found decreased overall survival with rs1077858. Prostate cancer cell lines carrying the risk allele for rs1077858 also exhibited an increased expression of SLCO2B1 (Wang *et al.* 2016). Due to small sample sizes (Wang *n*  = 68 and Yang *n*  = 135 patients, respectively), the findings in these studies still lack statistical power. Additionally, neither of these studies addressed the outcomes when SNPs multiple APUC genes are presented altogether in patients.

A subset of cytochrome p450 (CYPs) family of enzymes is involved in the production and conversion of androgens and other steroid hormones. Many studies examined the functional role of SNPs within families of CYPs involved in androgen production and their effect on outcomes in prostate cancer patients. Kanda *et al.* examined *CYP19A1*, which converts androgens into estrogens, a critical component of the sex hormone environment within men with mCRPC treated with ADT. They found that three specific SNPs (rs10459592, rs4775936, and rs2470152) when combined together created a higher ratio of estrone/androstenedione in a dose-dependent manner, and that these men had decreased risk of prostate cancer-specific mortality (Kanda *et al.* 2015).

*CYP17A1* encodes an enzyme that plays a key role in steroid hormone metabolism serving as a branch point between androgen and estrogen synthesis and is a target of the ADT abiraterone. *CYP17A1* SNPs have been implicated with prostate cancer risk. Previous literature has focused on a SNP *CYP17A1* rs743572 that lies within the untranslated promoter region and creates an additional transcription factor binding site, subsequently increasing the production of steroid hormone (Mononen & Schleutker 2009). However, there have been contradicting results on whether the WT (Gsur *et al.* 2001, Kittles *et al.* 2001, Yamada *et al.* 2013) or variant allele (Wadelius *et al.* 1999, Habuchi *et al.* 2000, Stanford *et al.* 2002, Antognelli *et al.* 2005) is associated with decreased risk. A meta-analysis involving over 2400 prostate cancer patients concluded that *CYP17A1* rs743572 SNP was not likely to significantly impact the risk for prostate cancer occurrence (Ntais *et al.* 2003). However, Yamada *et al*. recently found increased risk of progression to castration-resistant prostate cancer in Japanese men with prostate cancer treated with ADT with rs743572 *CYP17A1* (Yamada *et al.* 2013). Interestingly, Han *et al.* showed that among Korean men with prostate cancer, there were no significant correlations between rs743472 *CYP17A1* and prostate cancer mortality, metastatic potential, or histologic aggressiveness. They also performed haplotype analysis including 12 SNPs in *CYP17A1* to investigate associations with prostate cancer susceptibility. Here, they found a certain haplotype of *CYP17A1* was associated with prostate cancer mortality, while a different SNP, rs17115149* CYP17A1*, was associated with histologic aggressiveness and Gleason scores (Han *et al.* 2015).

Other CYPs such as *CYP3A4* have been examined in prostate cancer. Two decades ago, Rebbeck *et al.* identified a novel genetic variant *CYP3A4-V* that contains an A→G mutation in the 5’ upstream regulatory region of the *CYP3A4* gene. They found that the presence of this *CYP3A4-V* allele was associated with higher Gleason grade and higher TNM tumor staging at the time of diagnosis (Rebbeck *et al.* 1998). This was most notable in men diagnosed at age 64 or older and was primarily observed within White men. This result was subsequently corroborated in a cohort of Black men, where 176 men with prostate cancer homozygous for the *CYP3A4-V* variant had higher tumor grade and stage at the time of diagnosis, with increased significance in men who were diagnosed at the age of 65 or older (Paris *et al.* 1999). A separate case–control study of over 440 cases of prostate cancer (in a primarily White cohort) found that *CYP3A4-V* was associated with clinically aggressive disease (as based on Gleason grade and tumor stage) at time of diagnosis and inversely associated with less-aggressive disease at the time of diagnosis (Plummer *et al.* 2003). This inverse relationship with less aggressive disease was observed when examining multiple other *CYP3A4* SNPs as well as *CYP3A4* haplotype in a separate study (Loukola *et al.* 2004). There have been multiple reports of a specific SNP, *CYP3A4* rs680055, and prostate cancer risk after analysis and stratification for factors such as family history of prostate cancer, personal history of benign prostate hyperplasia (BPH), or cigarette smoking (Zeigler-Johnson *et al.* 2004, Stone *et al.* 2005, Rebbeck *et al.* 2008); however, these results were not found by Han *et al*. in their study of 240 Korean men (Han *et al.* 2015). These overall results suggest that the variant in the *CYP3A4* genotype is associated with increased prostate cancer tumor aggressiveness. Additionally, there are no studies as of now examining the role of this gene and its associated SNPs in metastatic prostate cancer and should be done moving forward given the high correlation of other APUC genes with outcomes in mCRPC and mCSPC alike.

The first conversion in the steroidogenesis pathway is performed by another CYP family enzyme, *CYP11A1*. This enzyme converts cholesterol into pregnenolone within the mitochondria of steroid-producing mammalian tissues and is a key function of the APUC pathway, as pregnenolone is subsequently converted into AR-activating substrates (Schwarz *et al.* 1997, Durocher *et al.* 1998, Franks *et al.* 1998). Previous studies have shown that the regulation of steroid hormone synthesis occurs due to transcriptional upregulation of *CYP11A1* (Moore *et al.* 1990). Additionally, *in vitro* analyses have revealed a (*tttta)*n-5 bp tandem repeat upstream of the translation initiation site of *CYP11A1* (Schwarz *et al.* 1997, Durocher *et al.* 1998, Franks *et al.* 1998) with the *CYP11A1 (tttta)4* being the shorter allele and *CYP11A1 (tttta)6* being the longer allele. The absence of this *CYP11A1 (ttta)4* shorter allele (and therefore homozygosity for longer *CYP11A1 (tttta)6* allele) is associated with hyperandrogenism and increased risk of polycystic ovarian syndrome (Gharani *et al.* 1997, Diamanti-Kandarakis *et al.* 2000). With this information in mind, Kumazama *et al.* examined the relationship of this *CYP11A1* polymorphism and prostate cancer. They found that there was no significant difference between the genotypic frequency for the presence of the *CYP11A1 (tttta)4* allele between prostate cancer patients and healthy controls. However, prostate cancer patients without the *CYP11A1 (tttta)4* allele had an increased risk of metastatic disease and increased risk of high-grade disease on biopsy (Gleason grade 8 or higher) when compared to prostate cancer patients with the *CYP11A1 (tttta)4* allele (Kumazawa *et al.* 2004). Their results suggest that the absence of this shorter allele and therefore homozygosity of the longer *CYP11A1 (ttta)6* allele is associated with more aggressive and advanced prostate cancer. The absence of an association between *CYP11A1 (ttta)4* and prostate cancer development was confirmed in another study by Cicek *et al.* (2005); however, they were not able to corroborate Kumazama’s findings in which lack of *CYP11A1 (tttta)4* was associated with increased stage and grade of prostate cancer. The authors suspected this was due to low sample size of high-stage prostate cancer. Douglas *et al.* examined a separate SNP *CYP11A1 rs2277602* resulting in a C→A polymorphism with C being the major allele and A being the minor allele and its association with prostate cancer. They found no evidence of an association of the presence of this polymorphism and prostate cancer (Douglas *et al.* 2005). A study on the genomic relationship between *CYP11A1* and prostate cancer using The Cancer Genome Atlas revealed that *CYP11A1* was significantly downregulated in prostate cancer (Fan *et al.* 2016). Subsequent genomic analysis of *CYP11A1* alone and in combination of other APUC genes is warranted. Aside from these studies, other works have shown this *CYP11A1 (ttta)4* polymorphism in an APUC gene to be associated with increased risk of breast cancer (Zheng *et al.* 2004), once again suggesting the importance of APUC genes in other steroid hormone-driven processes.

Work is also being done examining the interactions and functional relationship between SNPs between multiple APUC genes across different stages of androgen regulation and their impact on prostate cancer outcomes. Prizment *et al*. created a polygenic risk score as an unweighted sum of the risk alleles associated with higher androgen levels within *HSD3B1, SLCO2B1,* and 5-alpha reductase type 2 (*SRD5A2*). They found that higher scores were associated with a three-fold increased risk of prostate cancer mortality within 489 White men independent of stage or age (Prizment *et al.* 2021). These findings were not seen within men of Black ethnicity or when combining the two populations. These findings provide credence to the idea of a complex interplay between APUC genes, in which the aggregate of minor physiological effects within individual APUC variants may altogether yield significant biological activity in prostate cancer patients. In addition, APUC variants have different penetrance or exhibit mechanistic differences in a manner specific to ethnicity. Thus, the status of APUC genetic variants (Platz & Giovannucci 2004, Fujimoto *et al.* 2017) may distinctively regulate the higher prostate cancer mortality rates in Black men (http://cancerstatisticscenter.cancer.org/#!/). Altogether, these compelling observations warrant further consideration in additional patient studies that consider ethnicity.

Clearly we know that SNPs within many APUC genes have roles in both primary and metastatic prostate cancer as evidenced by both cell and tissue studies alike. However, more studies examining these other genes analogous to the large body of literature behind *HSD3B1* are currently required. These studies must consider treatment, stage, ethnicity, and status of other APUC genes. Together, this will paint a clearer picture of how this family of genes impacts survival of prostate cancer patients.

## Mechanistic studies on APUC genes

In addition to genetics and genomics, there is a growing and compelling story to be told about cellular and mechanistic effects of APUC genes within prostate cancer cells. Specifically, a growing body of research highlights the molecular functions in which APUC genes regulate tumorigenicity or other intracellular pathways.

The enzyme 3βHSD1 encoded by the APUC gene *HSD3B1* is the rate-limiting enzyme in the conversion of the adrenal-produced DHEA to the most potent AR ligand DHT. The story of SNPs causing differences in prostate cancer outcomes is well known; however, we also have an understanding of how this enzyme is playing a role intracellularly. At a population level, the ‘adrenal-permissive’ SNP 1245(A→C) within *HSD3B1* impacts prostate cancer outcomes. Within a cell, Chang *et al.* identified that this polymorphism creates a gain-of-stability mutation within the 3βHSD1 protein product by causing an asparagine (A) to be exchanged for threonine (T) at the 367 amino acid residue labeled as 367T. They showed that this (N367T) exchange within the 3βHSD1 protein product does not impact enzymatic catalytic function. However, this allows the enzyme to be resistant to both ubiquitination and degradation. This conferred what would represent a gain-of-stability mutation that significantly increased flux of DHEA to DHT when comparing LNCaP cells with (367T) vs LAPC4 cells with (367N) (Chang *et al.* 2013). Furthermore, this (N367T) change was found to be somatically selected for cells treated with abiraterone in tumor xenograft experiments. Targeted blocking of 3βHSD1 with RNAi inhibited the synthesis of DHT and AR response via target genes within LNCaP cells. Finally, they found that overexpression of 3βHSD1 (367T) phenotype accelerated the flux of DHEA to DHT within LAPC4 cells and shortened the time to development of CRPC xenograft tumors.

In CRPC, intratumoral androgen synthesis is considered a marker for androgen responsiveness. In laboratory studies, Hettel *et al.* found that *HSD3B1* transcription was induced in four separate CRPC cell lines after androgen induction, as opposed to androgen deprivation (Hettel *et al.* 2018). Protein levels of 3βHSD1 reflected these transcriptional increases in CRPC cell lines with both the WT and the adrenal-permissive *HSD3B1*. Furthermore, *HSD3B1* expression was reduced after initiation of castration with enzalutamide in a CRPC xenograft mouse model. All told, these results suggest a cell-intrinsic feed-forward positive regulation of *HSD3B1* by androgens in both cell line and *in vivo* models.

Finally, we highlighted that Sharifi *et al.* found compelling differences in CRPC patients with *HSD3B1* 1245(A→C) that received the CYP17A1 inhibitor abiraterone acetate, which blocks extra-gonadal androgen synthesis (Alyamani *et al.* 2018). Abiraterone is metabolized by 3βHSD1 into multiple metabolites, including the AR-activating 3-keto-5alpha-abiraterone metabolite. They showed that CRPC patients with *HSD3B1* 1245(A→C) had increased generation of this AR-stimulating metabolite, compared to those without the *HSD3B1* polymorphism, in a step-wise fashion. Clearly, 3βHSD1 has an integral role in the extra-gonadal synthesis of androgens in castration-resistant prostate cancer cells, and when we combine the results of these two studies, it suggests that *HSD3B1* genotype status impacts future strategies for pharmacologic treatment of CRPC, and that 3βHSD1 may be an actionable target for drug therapies.

Other than* HSD3B1*, the Sharifi group has also mechanistically studied the *SRD5A* family of genes in CRPC. Conventionally, it is thought that intratumoral production of DHT in CRPC patients requires a stepwise progression of androstenedione (AD) reduction to testosterone by 17BHSD, and subsequently, testosterone conversion to DHT via 5alpha-reduction by SRD5A (Scher & Sawyers 2005, Luu-The *et al.* 2008, Penning *et al.* 2008). When comparing benign prostate tissue to CRPC, increases in SRD5A1 expression over SRD5A2 drives its features as the dominant form of SRD5A enzyme (Titus *et al.* 2005, Stanbrough *et al.* 2006, Montgomery *et al.* 2008). It is conventionally thought that this upregulation drives CRPC progression due to SRD5A1 conversion of testosterone → DHT. However, both AD and testosterone are substrates for SRD5A in fresh prostatectomy tissues (Dai *et al.* 2017) and Chang *et al*. showed in a CRPC cell model that the primary route of DHT production is not through testosterone, but rather through SRD5A1 reduction of AD to 5alpha-androstendione and subsequent conversion into DHT (Chang *et al.* 2011). This result was seen in both CRPC cell lines and fresh tissue from human tumor metastases, and CRPC growth in mouse xenograft models was dependent on this pathway and SRD5A1 expression.

To elucidate cell-intrinsic functions of APUC genes, future studies may include extensive laboratory experi­ments or single-cell capable technologies that examine patient samples. Altogether, current mechanistic results support that the genetic and genomic status of APUC genes in a patient should be considered for the clinical management of CRPC patients. As examples, *HSD3B1* variant patients may have a greater benefit from specific forms of treatment intensification with upfront ADT (Hofmann *et al.* 2021). Clinicians and researchers should also consider the use of enzalutamide or apalutamide which potentially yield different metabolite profiles from abiraterone when *HSD3B1* variants are observed.

## Conclusion and future directions

As of 2022, there are a few promising actionable biomarkers such as AR-V7 (Antonarakis *et al.* 2014) that explain the variability in response against novel androgen-targeted therapies within prostate cancer. However, these markers cannot explain the total spectrum of patient responses, specifically those of patients on ADT/ART. According to the American Cancer Society, 70% of men with metastatic prostate cancer at diagnosis die within 5 years. This high rate of death may be attributed to continuing synthesis of intra-tumoral androgens and persistent activation of the AR pathway in metastatic prostate cancer despite chemical or surgical castration as well as potent AR inhibition. Increases in oncogenic AR activity may be driven by somatic changes that upregulate crucial APUC genes in the metastatic prostate cancer tumors, such as *HSD3B2* (1.8-fold increase) and *SRD5A1* (2.1-fold increase). Current literature (Penning *et al.* 2006, Montgomery *et al.* 2008, Mitsiades *et al.* 2012, Aggarwal *et al.* 2015) implicates APUC genes as upregulated mechanisms in prostate cancer, suggesting a need for a comprehensive characterization of the APUC landscape in metastatic prostate cancer. Additionally, as genomic tools and the access to tissue and patient genetic databases grow, there is a need to correlate both genetic differences in APUC genes with the downstream genomic and transcriptomic effects via deep sequencing techniques.

APUC genes, such as the *HSD3B1* genetic variants, may also interact with other tumor-promoting pathways such as the cell cycle (Chen *et al.* 2019, 2020). As gene editing tools that permit gene overexpression or gene ablation are becoming readily available, critical investigations should examine the relationship between APUC genes and other known genes and pathways that cause ADT/ART resistance, such as *FOXA1* (Adams *et al.* 2019, Parolia *et al.* 2019, Shah & Brown 2019), *FGFR, CDKs, MDM4,* and *CREB5* (Bluemn *et al.* 2017, Han *et al.* 2017, Hwang *et al.* 2019, Elmarakeby *et al.* 2021)*.* The functions of these APUC genes require further validation in multiple prostate cancer cell lines within the laboratory. These cell-based studies must also be combined with associative analyses between genetic and somatic alteration status of APUC genes, overall survival, and response status toward hormone therapy. All told, these studies will provide critical insights into the mechanisms through which APUC genes regulate survival of individuals with late-stage prostate cancer and may propose many possible therapeutic targets.

APUC genes also inform of differential genetics that regulate prostate cancer progression among distinct ethnicities. The current studies that exist examining APUC genes are primarily within cohorts of either entirely White or Asian men. As shown in Table 2, there are differences in the rates of certain APUC variants based on race such as *HSD3B1* rs1047303 (https://www.ncbi.nlm.nih.gov/snp/rs1047303) and *SLCO2B1* rs1789693 (https://www.ncbi.nlm.nih.gov/snp/rs1789693). Thus, the higher mortality rate of prostate cancer in Black men and low rate in Asian men (http://cancerstatisticscenter.cancer.org/#!/) could be partially explained by different population attributable risk associated with different prevalence of APUC genetic variants (Platz & Giovannucci 2004, Fujimoto *et al.* 2017). To fully characterize and understand this family of genes, a large cohort of equitable and well-represented racial and ethnic groups must be performed to understand this relationship to shed light on drivers of differences in prostate cancer outcomes, particularly for Black men.

**Table 2:** SNPs^a^ in two APUC genes.

Gene (chromosome) androgen-regulating function	SNP function	Allele		Frequency of major allele			
		Major	Minor	All	White men	Black men	Asian men
HSD3B1 (1) synthesis production	rs1047303 (1245C) missense	A	C^b^	0.8335	0.66	0.9145	0.9177
SLCO2B1 (11) transport and uptake	rs1789693 intron	A	T	0.4984	0.6958	0.2481	0.3948

^a^These two SNPs have been consistently linked with PC progression in clinical studies of PC patients. ^b^Risk allele is highlighted.

The studies we have presented support that the genetic or genomic interpretation of APUC gene status will contribute to understanding of a patient population that fall prey to this particular mechanism of resistance. Current data presented in this review suggest that polymorphisms in APUC genes mediate success or failure of ADT (abiraterone) or ART (enzalutamide) and may foster understanding of the disease and personalization around APUC biomarkers. Both single-nucleotide polymorphisms and tandem repeats within APUC genes have been shown to be associated with decreased overall survival, increased tumor burden, and decreased time to cancer progression in men with prostate cancer, primarily in men with mCRPC treated with ADT. Specifically, the large body of evidence behind the adrenal-permissive polymorphism in *HSD3B1* lends a possible target gene both diagnostically as well as therapeutically as we move forward. We have presented evidence in which other less studied APUC genes may have similar functions as *HSD3B1* both in a population and in tumor cells. While complex, the potential interactions between a combination of APUC genes and their individual polymorphisms may play an even larger role in the creation of a novel pathway within mCRPC and change our understanding of a new type of mCRPC. Studies that examine these APUC gene interactions within larger patient cohorts would create a clearer picture of the aggregate effect of APUC gene polymorphisms. Lastly, there are already currently available clinical and genetic tools and technologies to evaluate prostate cancer outcomes based on *HSD3B1, SLCO2B1,* and *SRD5A2* genotype and their relationships to disease, which will guide future biomarker-driven treatment.

Overall, the family of APUC genes and their respective genetic variants are known to impact prostate cancer outcomes of all kinds and most notably within the lethal mCRPC and mCSPC that lack therapeutic options upon failing ADT and ART. These findings and the current genetic and genomic tools available make it imperative to study this family of genes further. The future work described above has the potential to discover and classify a new understanding of a subtype of mCRPC and subsequently change the paradigm in how patients with this disease are treated. In the time of personalized medicine where genetic tests are becoming commonplace, this work will hopefully create new individualized care for men with advanced prostate cancer and ultimately provide improved outcomes.

## Declaration of interest

The authors declare that there is no conflict of interest that could be perceived as prejudicing the impartiality of this review.

## Funding

A Prizment, S Halabi, N Sharifi and C J Ryan received funding from NIH/NCI (R01 CA249279).
